# Serial Intervals and Incubation Periods of SARS-CoV-2 Omicron and Delta Variants, Singapore

**DOI:** 10.3201/eid2904.220854

**Published:** 2023-04

**Authors:** Kangwei Zeng, Santhya Santhya, Aijia Soong, Nitika Malhotra, Dhanushanth Pushparajah, Koh Cheng Thoon, Benny Yeo, Zheng Jie Marc Ho, Mark Chen I Cheng

**Affiliations:** National Centre for Infectious Diseases, Singapore (K. Zeng, Santhya, A. Soong, M. Chen I-Cheng);; Ministry of Health, Singapore (K. Zeng, N .Malhotra, D. Pushparajah, Z.J.M. Ho, M.C. I-Cheng);; KK Women's and Children’s Hospital, Singapore (K.C. Thoon);; National University of Singapore, Singapore (K.C. Thoon);; National Technological University, Singapore (K.C. Thoon);; National Public Health Laboratory, Singapore (B. Yeo)

**Keywords:** COVID-19, serial interval, incubation period, Omicron, Delta, variant of concern, coronavirus disease, SARS-CoV-2, severe acute respiratory syndrome coronavirus 2, viruses, respiratory infections, zoonoses, Singapore

## Abstract

We compared serial intervals and incubation periods for SARS-CoV-2 Omicron BA.1 and BA.2 subvariants and Delta variants in Singapore. Median incubation period was 3 days for BA.1 versus 4 days for Delta. Serial interval was 2 days for BA.1 and 3 days for BA.2 but 4 days for Delta.

The World Health Organization declared the SARS-CoV-2 Omicron variant a variant of concern on November 26, 2021 (*1*), and Omicron rapidly displaced Delta as the dominant variant. After detecting the first local Omicron case on December 9, 2021, the Singapore Ministry of Health performed rigorous contact tracing for the initial cases, which enabled us to compare serial interval and incubation periods of Omicron with similar investigations performed earlier for the Delta variant.

## The Study

We obtained case linkages, source exposure date, and date of first COVID-19–related symptom onset from the Ministry of Health contact tracing team for PCR-confirmed cases on the basis of previously described procedures for epidemiologic investigations (*2*). We also determined whether initially asymptomatic case-patients’ symptoms were subsequently reported through medical chart review.

We identified Delta variant cases from selected clusters with epidemiologic investigations during April 27, 2021–July 2, 2021, when Delta was dominant; 95% (n = 103) were confirmed by whole-genome sequencing (WGS). Omicron BA.1 cases were based on investigations beginning December 9, 2021. Of those, 67% (n = 66) were WGS-confirmed; 23% (n = 22) were not sequenced but exhibited spike (S) gene target failure on PCR, whereas 10% (n = 10) were presumed to be BA.1 on the basis of epidemiologic links. Omicron BA.2 cases were identified in investigations beginning January 3, 2022. Of those, 18% (n = 8) were WGS-confirmed; 26% (n = 12) were not sequenced but did not exhibit S gene target failure, whereas 50% (n = 23) were presumed to be BA.2 on the basis of epidemiologic links.

Among potential transmission pairs, we identified pairs with clear infector–infectee relationships where onset dates for both were available. Analysis of incubation period (time from exposure to onset) included only case-patients who acquired infection from a known source with exposure limited to 1 day. We based serial interval on the difference in onset dates within each transmission pair. We excluded transmission pairs with multiple possible transmission pathways within the household or social network and pairs (n = 17, 8%) with zero or negative serial interval because epidemiologic investigations suggest several of those pairs involved questionable onset dates. Serial interval was further stratified by whether transmission pairs originated from a household or nonhousehold setting. We report medians and interquartile ranges (IQRs) and assessed statistically significant differences using the Wilcoxon rank-sum test (Figure). We also estimated means and 95% CIs of serial interval and incubation period for each variant by fitting a Gamma and Weibull model using the maximum-likelihood method (Appendix). 

**Figure:**
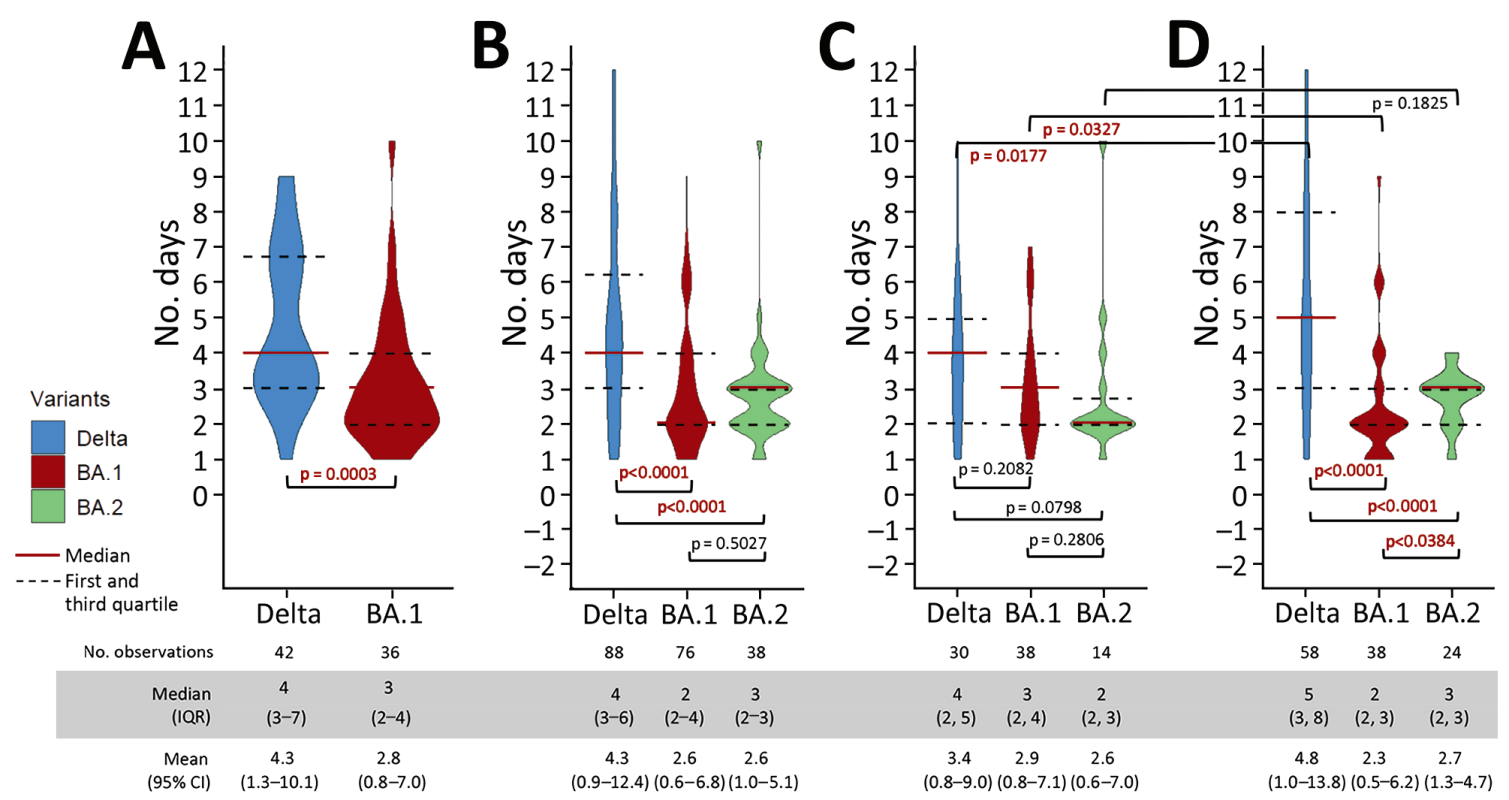
Comparative analysis of serial interval of SARS-CoV-2 Delta and Omicron variants, Singapore. A) Incubation period using only transmission pairs with clear epidemiological links and single-day source exposure to primary case. B) Serial interval of all symptomatic transmission pairs. C) Serial interval of all symptomatic transmission pairs with transmissions within households. D) Serial interval of all symptomatic transmission pairs with transmissions outside of households (workplace, social events). Means and 95% CIs are based on fitting a Gamma distribution using the maximum-likelihood method. p values were calculated using Wilcoxon rank-sum test.

Based on data for 36 transmission pairs for the Omicron BA.1 subvariant and 42 transmission pairs for the Delta variant (Table), the incubation period for BA.1 was shorter by ≈1 day, a median of 3 (IQR 2–4) days, compared with 4 (IQR 3–7) days for Delta (Figure). Incubation period could not be calculated for BA.2 because many cases lacked clear exposure dates.

**Table:** Demographic data for SARS-CoV-2 Omicron and Delta variants case-patients in study of incubation periods and serial intervals, Singapore*

Characteristic	Incubation period cases			Serial interval cases		
	Delta, n = 42	BA.1, n = 36		Delta, n = 88	BA.1, n = 76	BA.2, n = 38
Age group, y						
Median (IQR)	24 (9–34)	26 (21–38)		32 (21–41)	27 (20–42)	4 (3–33)
0–21	15 (35.7)	10 (27.7)		23 (26.1)	23 (30.3)	25 (65.8)
22–39	20 (47.6)	18 (50.0)		39 (44.3)	30 (39.5)	7 (18.4)
40–59	3 (7.1)	7 (19.4)		14 (15.9)	16 (21.1)	5 (13.2)
>60	4 (9.5)	1 (2.8)		12 (13.6)	7 (9.2)	1 (2.6)
Sex						
M	33 (78.6)	23 (63.9)		56 (63.6)	43 (56.6)	16 (42.1)
F	9 (21.4)	13 (36.1)		32 (36.4)	33 (43.4)	22 (57.9)
Ethnicity						
Chinese	26 (61.9)	21 (58.3)		51 (58.0)	35 (46.1)	21 (55.3)
Malay	13 (31.0)	1 (2.8)		26 (29.5)	7 (9.2)	10 (7.9)
Indian	1 (2.4)	4 (11.1)		1 (1.1)	15 (19.7)	3 (26.3)
Others	2 (4.8)	10 (27.8)		10 (11.4)	19 (25.0)	4 (10.5)
Vaccination status						
Unvaccinated	38 (90.5)	1 (2.8)		76 (86.4)	12 (15.8)	25 (65.8)
Partially vaccinated	1 (2.4)	0		4 (4.5)	0	0
Fully vaccinated	3 (7.1)	27 (75.0)		8 (9.1)	45 (59.2)	6 (15.8)
Boosted	0	8 (22.2)		0	19 (25.0)	7 (18.4)
Type of transmission						
Household	0	1 (2.8)		30 (34.1)	38 (50.0)	14 (36.8)
Nonhousehold	42 (100)	35 (97.2)		58 (65.9)	38 (50.0)	24 (63.2)

*Values are no. (%) except as indicated.

For serial interval, we identified 76 transmission pairs for Omicron BA.1, 38 for BA.2, and 88 for Delta. BA.1 transmission pairs included 2 large clusters originating in a fitness center (n = 20 pairs) and a restaurant (n = 16), in addition to subsequent transmission chains to household members, social contacts, and coursemates in a training session. Other local BA.1 transmission pairs originated either from imported cases (n = 27) or unlinked local primary cases (n = 13) who then infected household and social or work contacts. BA.2 transmission pairs (n = 38) included 4 clusters from preschools/childcare centers and 14 household transmission pairs. Delta transmission pairs were selected from various clusters: of 2 social (n = 11), 3 workplace (n = 27), 2 nosocomial (n = 24), and 1 preschool (n = 26).

For BA.1, the median serial interval of 2 (IQR 2–4) days was shorter than its incubation period of 3 days and shorter than the serial interval for Delta (median 4 [IQR 3–6] days). For Delta, serial interval was similar to its incubation period (Figure). Of note, the difference in serial interval between BA.1 and Delta was greater for nonhousehold transmission. Moreover, although serial interval for BA.1 was shorter in the nonhousehold setting than the household transmission setting, the reverse was true for Delta. One possible explanation is that transmission of BA.1 to nonhousehold contacts occurs during presymptomatic and early symptomatic phases, before a person self-isolates, whereas some BA.1 patients recovering at home would continue to infect other household members over multiple days after symptom onset. In contrast, the Delta case-patients included in our analyses were isolated from household members upon diagnosis, which would reduce household transmission in the days after onset. As for BA.2, the median serial interval of 3 (IQR 2–3) days was significantly shorter than the Delta serial interval and slightly but not significantly longer than the serial interval for BA.1. Median nonhousehold serial interval of 3 (IQR 2–3) days for BA.2 was significantly longer than for BA.1. Whether this difference reflects changes such as reduced contact tracing for Omicron subvariants after January 2022 is unclear.

## Conclusions

The serial interval we observed for BA.1 is corroborated by other international data, including 1.5–3.2 days in England (S. Abbott et al., unpub. data, https://www.medrxiv.org/content/10.1101/2022.01.08.22268920v1; H. Allen et al., unpub. data, https://www.medrxiv.org/content/10.1101/2022.02.15.22271001v1) and 2.22 days in South Korea (*3*). Norway reported a similar median Omicron incubation period of 3 days (*4*). A study from Hong Kong, despite a small sample size (n = 13), similarly reported the estimated mean serial interval of BA.2 as 2.7 days (median 2.5 [SD 1.5] days) (*5*), although a study from Spain reported an incubation period of 3.1 days and longer serial interval of 4.8 days (*6*). Relaxing nonpharmaceutical interventions would cause longer serial intervals and might explain some of these differences. Given a serial interval shorter than its incubation period, Omicron might be more predisposed to causing transmission before symptom onset than Delta. As a consequence, isolation after symptom onset would be less effective at preventing transmission. The short serial interval also poses challenges for contact tracing to identify and isolate secondary cases before they become infectious. Strategies such as preevent testing and regular testing of at-risk persons must also be performed at shorter intervals to remain effective.

The first limitation of our study is that most BA.1 case-patients (87%) were fully vaccinated, whereas most BA.2 and Delta case-patients were not (BA2, 65% unvaccinated; Delta, 80% unvaccinated). More than half of BA.2 cases were from preschool or school clusters, so many case-patients were younger and unvaccinated. We therefore could not determine whether differences in serial interval and incubation periods were caused by vaccination status rather than an intrinsic property of the virus. Selection bias caused by cluster control strategies might also result in overrepresentation of cases from larger clusters and thus more cases from superspreading events, which might also be associated with shorter serial interval and incubation period. However, other than vaccination status, most of those biases apply to both Delta and Omicron and are therefore unlikely explanations for differences between Delta and the 2 Omicron subvariants.

Mean serial intervals of 2–3 days have been reported for some other human coronaviruses and influenza A, which are also predominantly infections of the upper respiratory tract (*7*). The short serial interval is hence consistent with laboratory experiments demonstrating that Omicron grows more rapidly than Delta in human nasal epithelial cultures (T.P. Peacock et al., unpub. data, https://www.biorxiv.org/content/10.1101/2021.12.31.474653v2).

In conclusion, Omicron BA.1 and BA.2 had shorter serial intervals than the Delta variant, which could partially explain Omicron’s more rapid epidemic trajectory. The shorter serial interval could also have rendered public health interventions for earlier COVID-19 variants less effective for Omicron subvariants.

Appendix Additional information about serial intervals and incubation periods of SARS-CoV-2 Omicron and Delta variants, Singapore.
