# Supplementary material for: Serial Intervals and Incubation Periods of SARS-CoV-2 Omicron and Delta Variants, Singapore
Source: Emerg Infect Dis. 2023 Apr;29(4):814–7. doi: 10.3201/eid2904.220854 (PMC10045676; doi:10.3201/eid2904.220854)
Supplement: Appendix — Additional information about serial intervals and incubation periods of SARS-CoV-2 Omicron and Delta variants, Singapore. [file 22-0854-Techapp-s1.pdf]

Article DOI: https://doi.org/10.3201/eid2904.220854

EID cannot ensure accessibility for supplementary materials supplied by authors. Readers who have difficulty accessing supplementary content should contact the authors for assistance.

Serial Intervals and Incubation Periods of SARS-CoV-2 Omicron and Delta Variants, Singapore

Appendix

Appendix Table. Parameters of Gamma, Weibull, and Normal fitted distribution for incubation period and serial interval of Delta, BA.1, and BA.2 variants*

Category	Variant	Model fitted	Median	95% CI	AIC	BIC
Incubation Period	Delta	Gamma	4.33	1.34 to 10.10	185.43	188.90
		Weibull	4.69	0.40 to 8.98	188.94	192.42
		Normal	4.53	1.08 to 9.36	185.19	188.66
	BA.1	Gamma	2.80	0.77 to 6.99	133.95	137.12
		Weibull	3.08	−0.48 to 6.64	149.13	152.30
		Normal	2.86	0.47 to 7.11	138.52	141.69
Serial Interval	Delta	Gamma	4.31	0.86 to 12.44	425.05	430.01
		Weibull	4.92	−0.86 to 10.70	444.20	449.16
		Normal	4.49	0.68 to 11.69	425.55	430.51
	BA.1	Gamma	2.58	0.63 to 6.76	274.46	279.13
		Weibull	2.87	−0.55 to 6.28	304.06	308.72
		Normal	2.65	0.41 to 6.75	281.60	286.26
	BA.2	Gamma	2.64	0.95 to 5.08	122.15	125.42
		Weibull	2.82	−0.06 to 5.69	140.93	144.21
		Normal	2.65	0.52 to 6.08	131.63	134.90
Serial Interval (Household Transmission)	Delta	Gamma	3.41	0.83 to 9.00	127.94	130.74
		Weibull	3.80	−0.29 to 7.89	133.31	136.11
		Normal	3.55	0.63 to 8.47	128.28	131.08
	BA.1	Gamma	2.90	0.81 to 7.14	142.64	145.92
		Weibull	3.18	−0.15 to 6.51	152.13	155.40
		Normal	3.01	0.58 to 6.92	145.05	148.32
	BA.2	Gamma	2.62	0.62 to 7.00	54.84	56.12
		Weibull	2.93	−1.36 to 7.21	65.63	66.91
		Normal	2.61	0.31 to 7.64	57.62	58.90
Serial Interval (non-Household Transmission)	Delta	Gamma	4.83	0.98 to 13.82	293.79	297.92
		Weibull	5.50	−0.69 to 11.69	302.00	306.12
		Normal	5.06	0.82 to 12.73	292.72	296.84
	BA.1	Gamma	2.27	0.52 to 6.16	132.30	135.57
		Weibull	2.55	−0.83 to 5.94	153.38	156.66
		Normal	2.31	0.31 to 6.38	137.32	140.59
	BA.2	Gamma	2.66	1.29 to 4.74	64.70	67.06
		Weibull	2.75	1.23 to 4.27	60.02	62.37
		Normal	2.77	1.26 to 4.14	59.27	61.63

*Normal distribution was fitted for comparison. Gamma fitted distribution was observed to be the overall best fit, with the lowest AIC and BIC for most fittings.

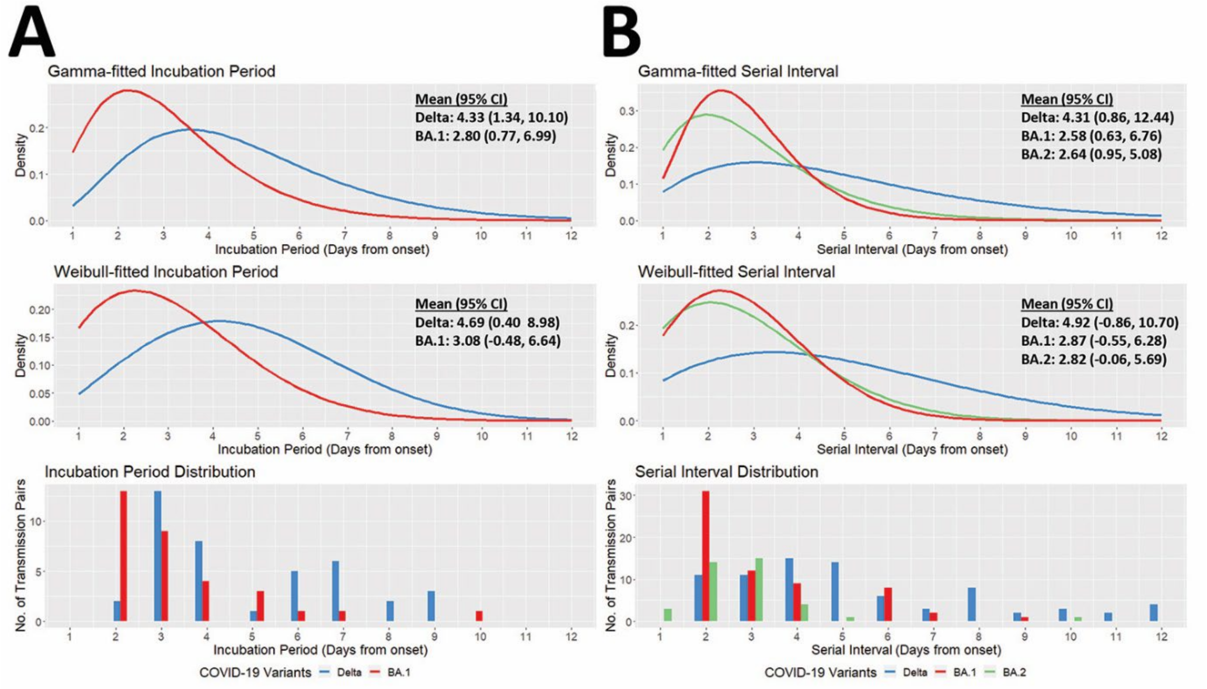

Appendix Figure 1. Gamma and Weibull distributions were fitted for a) incubation period and b) serial interval of Delta, BA.1, and BA.2 variants.

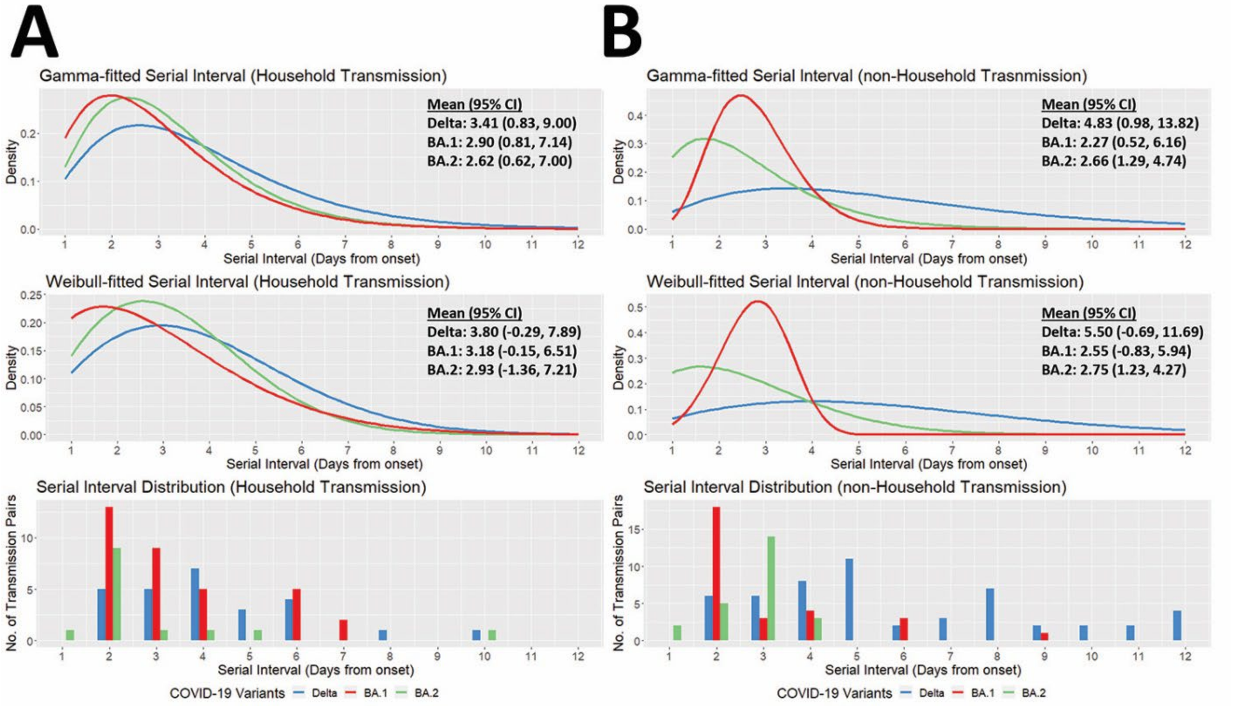

Appendix Figure 2. A) Incubation period and B) serial interval were further stratified into household and nonhousehold transmission settings.
